# Comprehensive Study of Drug-Induced Pruritus Based on Adverse Drug Reaction Report Database

**DOI:** 10.3390/ph16101500

**Published:** 2023-10-21

**Authors:** Yuriko Nakao, Mizuho Asada, Yoshihiro Uesawa

**Affiliations:** Department of Medical Molecular Informatics, Meiji Pharmaceutical University, 2-522-1 Noshio, Kiyose 204-8588, Tokyo, Japanmizuho@my-pharm.ac.jp (M.A.)

**Keywords:** drug-induced pruritus, itch, adverse events, mechanism, FAERS, comprehensive, signal detection, ROR, hierarchical clustering, principal component analysis

## Abstract

Drug-induced pruritus triggers a desire to scratch, thereby diminishing one’s quality of life. Certain instances of this phenomenon follow complex mechanisms of action that diverge from histamine-mediated pathways, known contributors to pruritus. However, investigations into the relationship between drugs and pruritus are limited. In this study, data mining techniques were employed to comprehensively analyze the characteristics of drugs linked to pruritus, using the FDA’s Adverse Event Reporting System (FAERS) data. Reports linked to pruritus demonstrated noteworthy differences in gender, age, and weight when compared with non-pruritus cases. Among the leading candidates for drugs prompting pruritus were ophthalmic drugs, systemic antibacterials, contrast media, dermatological antifungals, and dermatological preparations. A principal component analysis showed that the second principal component served as an indicator for distinguishing between onsets at mucous membranes or the skin’s surface. Additionally, the third principal component functioned as an indicator for categorizing administration methods as either invasive or noninvasive. Furthermore, a hierarchical cluster analysis conducted on these obtained principal components revealed the potential for classifying drugs based on the site of pruritus onset and the method of drug administration. These findings contribute to the development of targeted prevention and treatment strategies for avoiding pruritus in clinical practice.

## 1. Introduction

Drug-induced pruritus is characterized by an instinctive urge to scratch, brought about by a diverse array of drugs. This condition constitutes approximately 5–10% of all documented drug-related adverse effects and has garnered increasing attention in recent years [1,2]. Pruritus, commonly known as itching, has been reported to reduce the overall quality of life [3]. Pruritus is generally reported to increase the psychological, social, and economic burden of patients [4]. Itching can reduce quality of life and cause psychological distress, including sleep disturbances, difficulty concentrating, anxiety, and depression [3]. Itching can also affect appearance and grooming, leading to social isolation and discrimination [5,6]. In addition, itching increases the risks of skin damage and infections, and it carries an economic cost related to treatment and lost workdays [4,7]. Therefore, avoiding drug-induced pruritus is expected to improve all aspects of a patient’s quality of life. Furthermore, it can foster an inadequate adherence to prescribed drug regimens. The imperative to mitigate pruritus stems from its potential to heighten the likelihood of patients discontinuing treatment, thereby exacerbating the primary disease. Consequently, understanding the mechanisms behind drug-induced pruritus assumes paramount significance in steering effective clinical management and therapeutic choices.

Over the preceding decades, numerous studies have delved into the intricacies of drug-induced pruritus and identified various drugs as potential triggers. Commonly cited associated drugs include opioids, antimalarials, and certain antibiotics [1,8]. The reported occurrence of drug-induced pruritus demonstrates variation according to the pharmacological classification of the drug in question. Particularly, relative differences in incidence manifest in antimicrobials due to their diverse pharmacological mechanisms [9]. However, the classification of pruritus based on mechanisms remains predominantly unexplored.

Conversely, the mechanism of action of drug-induced pruritus can be unraveled from antipruritic drugs. Antihistamines, serving as the primary antipruritic drugs, inhibit histamine release from mast cells. Antihistamines are classified into two categories, namely H1 and H2, depending on the receptor on which they act [10]. Because histamine-induced pruritus occurs via H1 receptors, H1 antihistamines are generally effective against pruritus associated with urticaria and other conditions, whereas H2 antihistamines are generally considered ineffective against pruritus. Therefore, pruritus, primarily caused by histamine, is found to involve H1 receptors. However, pruritus that is not suppressed by H1 antihistamines is most likely caused by chemical mediators other than histamine [11]. In fact, certain pruritus-inducing drugs have been associated with multifaceted mechanisms involving several chemical mediators [12].

Investigating such complex phenomena requires the application of more advanced analytical methods when dealing with large databases. While some pioneering investigations have used data mining techniques to explore potential trends within adverse event databases [13], research employing this approach to explore drug-induced pruritus remains limited. Despite the growing importance of investigating pruritus, many studies pertaining to this condition have grappled with limitations such as a limited subject pool, data derived from a single country, and a confined concentration on specific drug-pruritus associations [1,14,15,16].

Hence, the present study undertook an all-encompassing, worldwide investigation into drug-induced pruritus. The US Food and Drug Administration’s (FDA) FAERS was used to conduct a comprehensive analysis to study suspected drug-induced adverse events on a global scale. This endeavor not only comprehensively investigated a broad spectrum of drug categories but also incorporated diverse patient characteristics (gender, age, and weight) and their association with pruritus. This study focused on the pharmacological classification of drugs known to induce pruritus and also aimed to identify drugs with a high risk of onset of pruritus whose relationship to pruritus is not yet established.

Findings from this study offer valuable insights into effective approaches for mitigating drug-induced pruritus, which may help contribute to the development of therapeutic strategies. An effort was exerted to examine patient demographics to enable doctors to enhance treatment choices by considering distinct patient characteristics. In addition, our objective was to provide an evaluation list of drugs to healthcare providers, warranting careful consideration when treating patients with an increased susceptibility to pruritus. We also aimed to contribute toward refining the precision of pruritus risk evaluation by classifying causative drugs based on their characteristics.

## 2. Results

### 2.1. Data Table for Analysis

**Figure 1:**
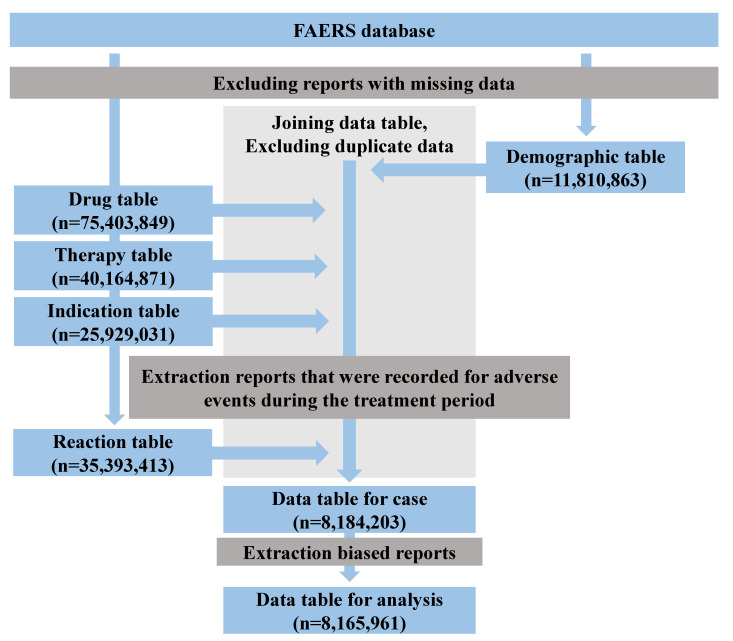
Flowchart of the data table creation.

Data extracted from FAERS encompassed records spanning from the first quarter of 2004 to the first quarter of 2020. The total number of adverse events obtained from the Demographic table totaled 11,810,863. Utilizing the procedural sequence outlined in the flowchart for constructing data tables, we employed the Drug table, comprising 75,403,849 reports, the Therapy table, encompassing 40,164,871 reports, and the Indication table with 25,929,031 reports (Figure 1). This chronological organization facilitated the identification of 8,184,203 reports that exhibited an apparent correlation between drug use and adverse drug reactions. Among these, 90,976 (1.1%) reports pertained to pruritus. Subsequent to the removal of reports exhibiting a pronounced potential for bias, an analysis-ready data table comprising 8,165,961 entries was employed.

### 2.2. Relevance of Drug-Induced Pruritus to Patient Characteristics

All patient groups, including both male-only and female-only cohorts, underwent scrutiny to ascertain the presence or absence of pruritus, along with considerations of age and weight (Table 1). For each parameter, data tables were subjected to analysis after eliminating reports with missing values.

In the comprehensive data table, pruritus was reported in 86,989 out of 7,396,343 cases (Table 1). Among these, the mean age within the pruritic and non-pruritic groups was 50.30 ± 20.24 and 53.17 ± 21.09 years, respectively. Notably, the pruritic group exhibited a statistically significant younger age (*p* < 0.0001), albeit with a negligible difference of 3 years. The mean body weights in the pruritic and non-pruritic groups were 88.26 ± 44.93 kg and 83.44 ± 44.55 kg, respectively, and while a significant difference was observable (*p* < 0.0001), the numerical 5 kg difference may not be considered clinically or practically significant although it is statistically significant.

Within the male data table, occurrences of pruritus were reported in 26,081 out of 2,752,150 cases (Table 1). The mean age for the pruritic and non-pruritic groups was 52.13 ± 20.65 and 55.57 ± 21.11 years, respectively. In essence, the non-pruritic group displayed statistically significant seniority (*p* < 0.0001), yet this translated to a marginal difference of 3 years. The mean weights for the pruritic and non-pruritic groups were 91.27 ± 44.77 kg and 86.22 ± 45.34 kg, respectively. Although a statistically significant difference was evident (*p* < 0.0001), the difference was insignificant at 5 kg.

Within the female data table, instances of pruritus were reported in 55,149 out of 4,089,839 cases (Table 1). Among these, the mean age for the pruritic and non-pruritic groups was 49.44 ± 19.99 and 55.15 ± 20.92 years, respectively. Similar to the male group, the non-pruritic group was notably older (*p* < 0.0001), although this difference was insignificant at 5 years. The mean body weights within the pruritic and non-pruritic groups were 86.63 ± 44.94 kg and 81.87 ± 43.90 kg, respectively. These were significantly different (*p* < 0.0001), yet the difference remained insignificant at 5 kg.

### 2.3. Signal Detection of Pruritus-Inducing Drugs

**Figure 2:**
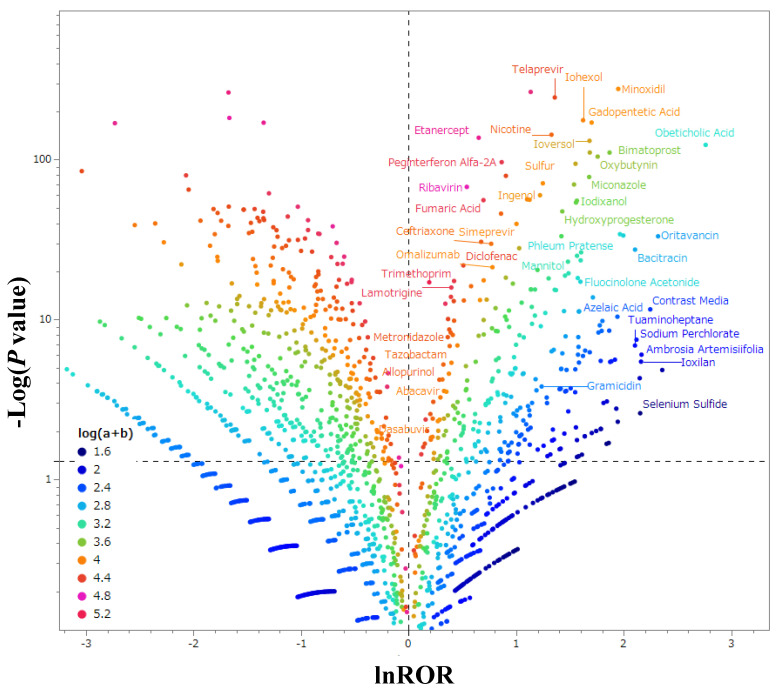
Drugs associated with pruritus.

A scatterplot was generated, positioning the natural logarithm of the reporting odds ratio (ROR) (ln(ROR)) along the *X*-axis and Fisher’s exact probability test (−Log 10 (*p*-value)) along the *Y*-axis (Figure 2). In the context of this plot, the positive X direction illustrates the distribution of drugs where pruritus occurrences surpass other adverse events. Similarly, the positive Y direction indicates notable statistical significance. In essence, the plots distributed in the upper-right corner of Figure 2 tend to significantly associate with pruritus. These drugs were classified based on their effects in accordance with the Anatomical Therapeutic Chemical Classification (ATC) of the Kyoto Encyclopedia of Genes and Genomes (KEGG), developed by Minoru Kanehisa [17,18,19]. The main medicinal classifications encompassing drugs exhibiting signals associated with cases of pruritus induction included ophthalmic drugs, systemic antibacterials, contrast media, dermatological antifungals, other dermatological preparations, corticosteroids and dermatological preparations, gynecological anti-infectives and disinfectants, antineoplastic drugs, immunosuppressive drugs, and vaccines (Table 2).

This figure depicts the relationship between pruritus and the suspected drug. The dotted line extending vertically along the *Y*-axis represents lnROR = 0, while the dotted line extending horizontally along the *X*-axis corresponds to *p* = 0.05. The color of each point is categorized based on the ordinary logarithm of the number of reports for each drug. Shades ranging from blue to green to red signify the ascending order of report counts, from the least to the most reported.

Illustrated in the upper-right quadrant of Figure 2 are drugs with notable signals. Across each drug category, the top five drugs with the highest reporting odds ratios were oritavancin (10.15), ioxilan (8.66), selenium disulfide (8.59), minoxidil (7.00), and bimatoprost (6.46).

Additionally, the drugs with the largest reported odds ratios without belonging to one of the main drug classes included obeticholic acid (15.79).

### 2.4. Characteristics of the Adverse Events Related to Pruritus Using Principal Component Analysis

The principal components’ contribution consisted of 29.5% for the first, 12.7% for the second, and 10.9% for the third. The contribution ratio signifies the proportion of each principal component’s variance relative to the total variance [20]. Subsequently, the relationship between the recommended terms linked to pruritus and the principal components was depicted in a plot featuring a loading vector for each recommended term.

Score plots and loading plots were generated, positioning the first principal component on the *X*-axis and the second principal component on the *Y*-axis. All vectors representing recommended pruritus-related terms on the *X*-axis demonstrated a positive correlation. Additionally, in the univariate analysis, the first principal component exhibited a negative correlation with the ordinary logarithm of the number of reports (a + b). Conversely, the primary drug classes positively correlated with the first principal component encompassed systemic antibacterials, anticancer drugs, ophthalmic drugs, immunosuppressive drugs, and systemic antivirals (Table 3). Drugs exhibiting a strong positive correlation with the first principal component, along with their corresponding factor loadings, included the contrast agent iopromide (9.40), the systemic antihistamine loratadine (4.71), the systemic antibacterial agent ceftriaxone (4.34), the systemic antibacterial and ophthalmic agent moxifloxacin (4.26), and the anti-inflammatory and anti-rheumatic, gynecological and topical for joint and muscle pain naproxen (4.22). Factor loadings are correlation coefficients that express the strength of the correlation between the principal components and individual variables [10]. A higher absolute value indicates a stronger correlation.

**Figure 3:**
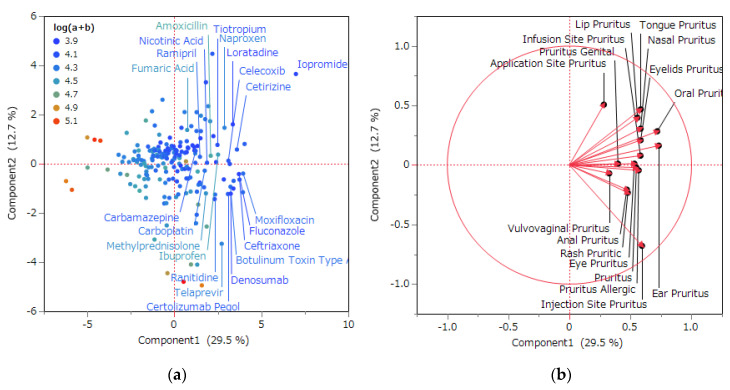
Relationship between pruritus-related adverse effects and drugs through a principal component analysis. (**a**) Score plot and (**b**) loading plot. (**a**) The score plot illustrates the correlation between drugs and the initial and second principal components. Each data point corresponds to a specific drug. (**b**) Each loading vector signifies a particular side effect. The length of the loading vector reflects the degree of correlation between side effects and the principal components.

This table presents the drugs within the plot that were positively correlated with the first principal component featured in the (a) score plot depicted in Figure 3. The arrangement is organized by drug effects according to the ATC of the KEGG.

**Figure 4:**
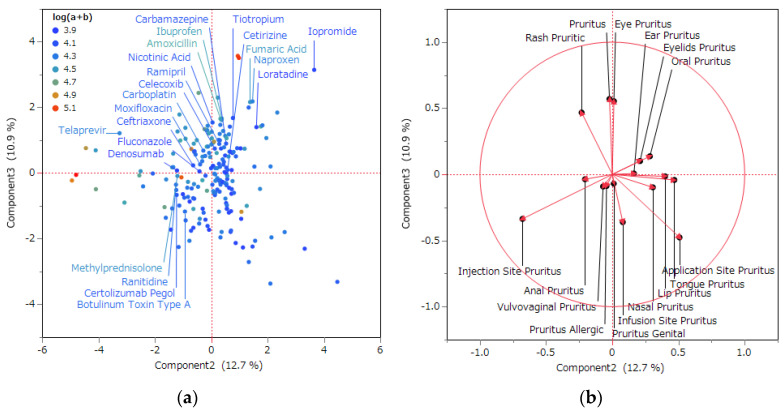
A principal component analysis of the relationship between pruritus-related adverse effects and drugs. (**a**) Score plot and (**b**) loading plot. The (**a**) score plot illustrates the correlation between each drug and the second and third principal components. Each data point corresponds to a specific drug. (**b**) Each loading vector denotes specific side effects. The magnitude of the loading vector reflects the intensity of the correlation between side effects and the principal components.

Subsequently, score and loading plots were generated, positioning the second principal component on the *X*-axis and the third principal component on the *Y*-axis (Figure 4). The adverse effects and their respective factor loadings associated with each variable that correlated positively with the second principal component included application site itchiness (0.50767), tongue itchiness (0.46828), lip itchiness (0.39825), nose itchiness (0.30621), and oral itchiness (0.28397). Conversely, the adverse effects and their factor loadings linked to variables negatively correlated with the second principal component comprised injection site itch (−0.67840) and erythema scratchiness (−0.23182).

In relation to the third principal component, the adverse effects and their associated factor loadings for each variable positively correlated with the third principal component encompassed itching (0.56996), itchy eyes (0.55315), and itchy erythema (0.46732). Conversely, the adverse effects and their factor loadings linked to variables negatively correlated with the third principal component included application site itching (−0.47643), injection site itching (−0.36073), and injection site itching (−0.33621).

### 2.5. Characteristics of the Adverse Events Related to Pruritus Using Hierarchical Clustering

**Figure 5:**
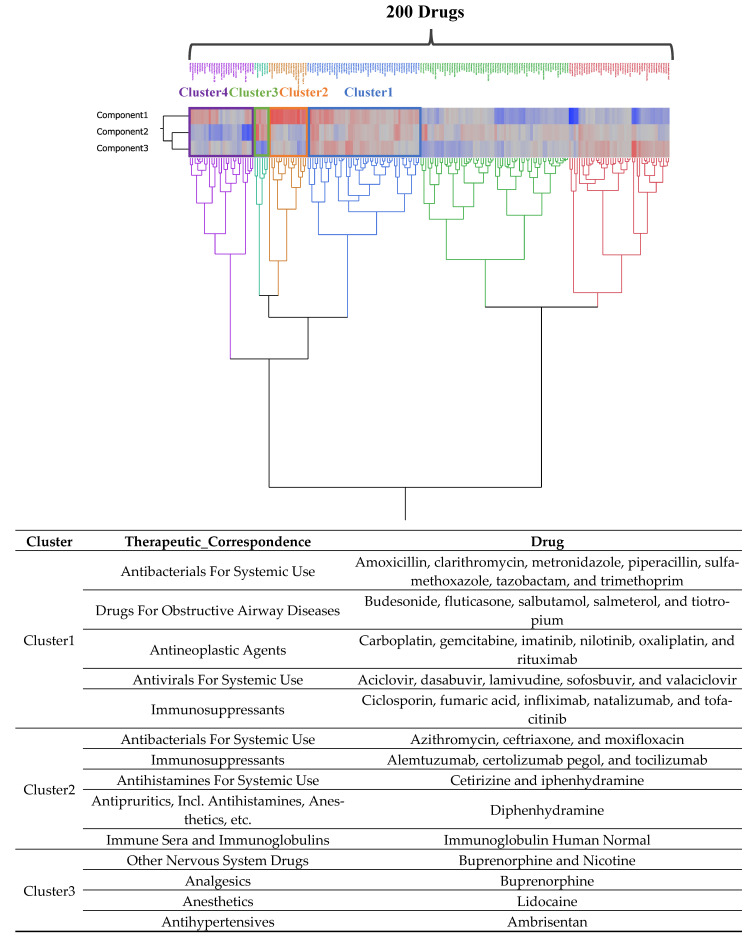

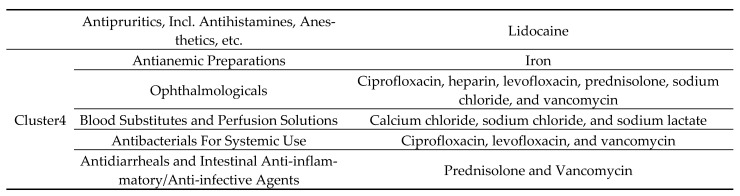
Classification of drugs related to principal components using a hierarchical cluster analysis.

From the hierarchical cluster analysis’s dendrogram, six distinct clusters emerged. Of these, we extracted drugs that were correlated with the principal components (Figure 5).

Within Cluster 1 (marked in blue boxes), systemic antibacterials and drugs for obstructive airway disorders, antineoplastics, systemic antivirals, and immunosuppressants were positively correlated with the first three principal components.

Within Cluster 2 (marked in orange boxes), immune sera and immunoglobulins, including systemic antibacterials and immunosuppressants, systemic antihistamines, antipruritics/antihistamines, and anesthetics, were positively correlated with the first principal component and negatively correlated with the second principal component.

Within Cluster 3 (marked in green boxes), other neurological drugs, oropharyngeal preparations, ophthalmic drugs, vasoprotective drugs, and antihypertensive drugs were positively correlated with the first and second principal components but negatively correlated with the third principal component.

Within Cluster 4 (marked in the purple box), anti-anemia preparations, ophthalmic drugs, blood substitutes and perfusate, antidiarrheals/intestinal anti-inflammatory and anti-infective drugs, and systemic antibacterial drugs were positively correlated with the first principal component and negatively correlated with the second principal component.

The hierarchical tree diagram depicts the interrelation among drugs linked to the initial three principal components. The diagram is divided into six clusters, delineated by dashed lines. The color gradient segregates the loading values of the principal components into a spectrum from red to gray to blue, denoting ascending levels of loading values.

Cluster 1 designates drugs displaying positive correlations with the first three principal components. Cluster 2 designates drugs displaying positive correlations with the first principal component but negative correlations with the second principal component. Cluster 3 designates drugs displaying positive correlations with the first and second principal components but negative correlations with the third principal component. Cluster 4 designates drugs displaying positive correlations with the first principal component but negative correlations with the second principal component.

## 3. Discussion

### 3.1. Definition of Pruritus

In a general context, drug-induced pruritus is typically characterized by primary itchiness devoid of skin lesions. It comprises approximately 5% of all reported adverse drug reactions [21]. Conversely, drug-induced pruritus accompanied by skin lesions is termed secondary itching. This phenomenon is acknowledged to stem from a detrimental cycle in which itching triggers scratching behavior, resulting in skin lesions, which subsequently induce fresh itching. Discriminating between these two forms of itching is notably challenging, and past research endeavors have often amalgamated both types of itching under a broader definition. In this current study, the correct identification of the presence or absence of skin lesions for each case was unattainable. Therefore, pruritus in the present investigation was delineated by encompassing both primary and secondary forms of itchiness.

### 3.2. Drug-Induced Pruritus and Patient Characteristics

Patients who reported experiencing pruritus were frequently observed to be of the female gender, significantly younger, and with a higher body weight compared to non-pruritic patients. Similar results were also evident in the context of pruritus associated with psoriasis [22]. An earlier study by Anil Gulsel Bahali et al. conducted a retrospective cross-sectional analysis involving 880 patients with psoriasis, revealing a notably higher prevalence of pruritus among women. Among the female participants, 324 exhibited pruritus while 160 did not (*p* = 0.005); for males, 229 experienced pruritus and 167 did not. The psoriasis patients with pruritus were noted to possess a considerably elevated Body Mass Index (BMI) in comparison to those without pruritus (a mean BMI of 28.2 ± 7.0 for pruritic individuals and 27.2 ± 6.3 for non-pruritic individuals; *p* = 0.025). However, Bahali et al.’s study yielded contrasting results concerning age in comparison to the present study. Their findings indicate that there was no significant age disparity between psoriasis patients with and without pruritus (the mean age of pruritic patients was 43.6 ± 16.7 years, and the mean age of non-pruritic patients was 44.2 ± 16.5 years, *p* = 0.649).

Their study focuses on pruritus as a symptom of a disease, unlike the current work that regards it as an adverse effect induced by drugs. Unfortunately, to our knowledge, there are no comprehensive epidemiological studies specifically on drug-induced pruritus. However, even though the mentioned study centers on pruritus in the context of a disease, it is believed to offer valuable insights into the epidemiology of pathological conditions that can inform our understanding of drug-induced pruritus. Other associated factors included the clinical type of psoriasis, depression, and systemic diseases. However, these factors had no significant correlation with pruritus. The study by Bahali et al. included fewer subjects than the present study, which could explain the differences in the associations of the factors with pruritus between the studies. By comparing previous research with the present study, we interpreted the risk factors for patients with pruritus. Patients with the aforementioned characteristics, such as young females with a higher body weight, were considered to have a high risk of developing pruritus, and they should therefore be administered drugs with a low risk of causing pruritus. However, their study did not describe the mechanism by which pruritus develops. A further investigation of this issue is warranted. Additionally, Bahali et al.’s research was retrospective and observational, which might have introduced biases in subject selection. Hence, there is a requirement for prospective follow-up studies, such as cohort studies, to ensure more accurate and reliable findings.

### 3.3. Characteristics of Pruritus-Inducing Drugs

In the present study, the primary drugs inducing pruritus encompassed ophthalmic drugs, systemic antibacterials, contrast media, dermatological antifungals, dermatological preparations, corticosteroids, gynecological anti-infectives and disinfectants, antineoplastics, and immunosuppressive drugs. Earlier studies also highlighted potential pruritogenic drugs, including antihypertensive drugs, anti-arrhythmics, anticoagulants, antidiabetic drugs, lipid-lowering drugs, antibiotics and chemotherapy, psychotropic drugs, antiepileptic drugs, contrast media, cell growth inhibitors, cytokine growth factors, monoclonal antibodies, and plasma bulking agents [1]. Among the drug classes reported in this study, antibacterial agents, contrast agents, and antifungal agents overlapped with the findings from these prior studies. Notably, hydroxyethyl starch (HES), despite having a confirmed pruritus mechanism, was not recognized as an inducer of pruritus in the current study [23].

Within the top five drug classes, the drugs with the most elevated RORs and their corresponding values were bimatoprost (6.46), oritavancin (10.15), ioxilan (8.66), selenium disulfide (8.59), and minoxidil (7.00). Furthermore, the single drug with the highest ROR value, which was not classified within the main drug classes, was obeticholic acid (15.79).

Bimatoprost is an eye drop employed for diminishing intraocular pressure in patients with open-angle glaucoma and ocular hypertension. Structurally resembling prostaglandin F2α, it functions as an agonist, activating the prostaglandin FP receptor. This stimulation leads to elevated levels of matrix metalloproteinases (MMPs) in the ciliary muscle and sclera. These MMPs facilitate the degradation of collagen within the extracellular matrix, thereby enhancing uveoscleral outflow and reducing intraocular pressure. To the best of our knowledge, no reports exist regarding the mechanism of action of bimatoprost that establish a connection with pruritus. Nevertheless, a four-year safety study has indicated that the bimatoprost ophthalmic solution triggers ocular pruritus in 9% of patients. For dermal applications, the incidence of ocular pruritus was reported to be below 4% [24].

Oritavancin diphosphate (Oritavancin) is a semi-synthetic, long-acting lipoglycopeptide (LGP) with potent activity against Gram-positive pathogens such as methicillin-resistant Staphylococcus aureus. It operates through three distinct mechanisms: (i) obstructing cell wall synthesis by binding to the peptide stems of peptidoglycan precursors, (ii) hampering the peptide transfer (cross-linking) phase of cell wall formation by binding to peptide cross-links within the cell wall, and (iii) rapid cell death attributable to the disruption of bacterial membrane integrity [25]. While our exploration did not reveal any reports suggesting a connection between pruritus and this mechanism of action, pruritus did emerge as an adverse event during a phase III international multicenter randomized double-blind clinical trial. In this clinical investigation conducted to evaluate the efficacy and safety of inpatient treatment for acute bacterial skin and skin structure infections, subjects were monitored for 60 days to assess the safety and tolerability of oritavancin, which has a prolonged distribution half-life. Notably, pruritus manifested as an injection-related reaction during the trial, which subsequently resolved upon reducing the injection rate [26].

Ioxilan is administered intravenously to facilitate excretory urography and contrast-enhanced computed tomography scans of both the head and body. This non-ionic monomeric substance possesses low osmolarity and was developed with the aim of enhancing the safety and tolerance of X-ray contrast agents [27]. To our knowledge, there are no reports on the mechanism of action of ioxilan that mention its association with pruritus. However, mild pruritus attributable to a contrast medium has been reported. We believe that minor side effects, including pruritus, have a short duration and generally do not require special treatment [28].

Selenium disulfide (SeS2) is an effective treatment option for dandruff, a milder manifestation of seborrhoeic dermatitis. It exhibits antifungal properties against Malassezia furfur and also demonstrates the ability to inhibit the growth of Staphylococcus epidermidis in vitro. SeS2-based shampoos reduce scalp dandruff, which has been linked to pruritus. In a randomized, double-blind, parallel-group study, participants who used a 1% salicylic acid shampoo containing selenium disulfide subsequent to an initial ketoconazole treatment experienced a decrease in pruritus (average score decreased from 2.16 to 0.81, *p <* 0.0004). Conversely, subjects who used selenium disulfide shampoo after an initial ketoconazole treatment displayed no additional reduction in pruritus compared to after the initial treatment (average score increased from 1.57 to 1.89, *p* = 0.3496). The observed variation in effectiveness is believed to arise from the concurrent presence of the keratolytic agent salicylic acid, which significantly reduced *Staphylococcus* spp. [29]. The results of this previous study suggest that SeS2 may not contribute to the reduction of pruritus on its own. Therefore, future intervention studies with and without SeS2 should determine its association with pruritus.

Topical minoxidil serves as a remedy for male and female pattern baldness, encompassing androgenetic alopecia [30]. Proposed mechanisms of action encompass vasodilation, anti-inflammatory properties, stimulation of the Wnt/β-catenin signaling pathway, and antiandrogenic effects. While our exploration yielded no reports linking pruritus to the mechanism of action, solutions containing minoxidil have previously been implicated in causing scalp pruritus. It has been mentioned that the incidence of pruritus caused by minoxidil is lower than that caused by polyethylene glycol [30]. Additional clinical trials have also established that topical minoxidil is primarily linked to scalp pruritus. The reason for this is believed to be the low absorption rate of minoxidil when applied topically, which leads to scalp inflammation and dandruff [30].

Obeticholic acid (OCA) is used in the secondary treatment of primary biliary cholangitis (PBC). OCAs have gained significance as a complementary approach to the standard utilization of ursodeoxycholic acid (UDCA) [31]. These OCAs target the endogenous farnesoid X receptor and exhibit a molecular structure akin to the ligand kenodeoxycholic acid. The mode of action involves (i) the inhibition of bile acid synthesis, (ii) the stimulation of the negative regulators of bile synthesis production, and (iii) the regulation of the bile acid transporter expression [31]. However, the exact reason behind OCA-induced pruritus remains elusive. A recent exploration ventured into the hypothesis that OCAs might trigger pruritus via the endogenous opioid system [32]. This hypothesis is supported by findings demonstrating that in experimental animals, accumulated bile acids due to bile stasis activate scratching behavior through TGR5 receptors, mediated by opioids [33]. Given that OCAs also interact with TGR5, it is suggested that OCA-induced pruritus is related to opioids. Indeed, in an international double-blind, placebo-controlled trial assessing OCA monotherapy in PBC patients, notable pruritus was reported in the OCA-treated group (placebo: 35%; OCA 10 mg: 70%; OCA 50 mg: 94%). A noteworthy proportion of patients in the OCA 10 mg group (15%) and the OCA 50 mg group (38%) discontinued treatment due to pruritus. These findings affirm the dose-dependent correlation between OCA and the incidence and intensity of pruritus.

On the other hand, many drugs have been reported to cause the adverse effects of pruritus, making it challenging to identify those that do not. However, the ROR indicated in this study is considered to be useful in estimating drugs with a relatively higher risk of inducing pruritus.

However, the lnROR might have been underestimated or overestimated because the number of reports (a + b) differed for each plot in the volcano plot. Therefore, we avoided a simple comparison of lnRORs by setting a lower limit for the number of reported cases. The substances found to induce pruritus included macrogol 400, polyethylene glycol, and propylene glycol (Table 2). FAERS registered a wide range of chemicals with health effects, including excipients, supplements, and cosmetics containing these compounds. The possibility of false positives is mentioned in the description section for antihistamines, but these excipients could have had significantly higher RORs because of the inclusion of false positives.

### 3.4. Principal Component Analysis

In this study, the relationship between each side effect (Preferred Term) and the corresponding drug was assessed using principal components. Each principal component was elucidated through a loading vector representing the associated side effects.

In the context of the first principal component, all vectors corresponding to side effects displayed a positive correlation (Figure 3b). Consequently, the first principal component was construed as an indicator of pruritus incidence.

Most drugs that correlated positively with the first principal component had approximate ordinary log((log(a + b))) values of the number of reports (Figure 3a). Therefore, we found that the RORs for these drugs were comparable. This comparison of RORs is likely to be less under- and overestimated than the comparison of RORs obtained using the volcano plot. In addition, each drug positively correlated with the first principal component, which could be interpreted as being positively correlated with the incidence of pruritus.

The drugs that exhibited a positive correlation with the first principal component, along with their respective drug classes, encompassed antihistamines such as loratadine (Figure 3a). Antihistamines have increasingly been utilized as antipruritics, although their efficacy has been reported to be inadequate for certain pruritus cases. This led to the speculation that the elevated frequency of antihistamine reports might arise from false-positive reports in pruritic patients who did not respond favorably to antihistamine treatments. Essentially, instances of residual or intensified pruritus might have been reported even when loratadine was administered as a therapeutic intervention.

Regarding the second principal component, the vectors representing adverse reactions were segregated into those that exhibited positive and negative correlations. Notably, strongly positively correlated adverse reactions encompassed sensations like itching at application sites, including the tongue, lips, nose, and oral cavity. Application site itching pertains to itching experienced on body surfaces, such as the skin and mucous membranes, where the drug is applied locally. This type of itching has been reported for various medications like ciprofloxacin and calcineurin inhibitors, particularly following topical applications, resulting in localized reactions on the skin and mucous membranes [1]. Furthermore, pruritus occurring in regions such as the tongue, lips, nose, and oral cavity is rooted in the mucosal epithelium [34]. Thus, it was postulated that these side effects share the characteristic of originating on mucous membranes. In contrast, adverse reactions that were strongly negatively correlated included injection site pruritus and erythematous pruritus. Since injection sites are classified based on the administration mode, such as epidermal, dermal interstitium, subcutaneous tissue, muscle, and vein, it was inferred that their impact on mucous membranes would be minimal [35,36]. For instance, skin reactions at injection sites occurred in about 10.3% of cases following COVID-19 vaccination, often accompanied by pruritus [37]. Erythema pruritus is a clear indication of the presence of a rash or skin lesion. Immune checkpoint inhibitor-associated skin toxicity, involving xerosis and pruritus followed by scratching (seen in 10–30% of patients), also frequently involves the appearance of a rash [38]. Consequently, it was assumed that these side effects share the trait of manifesting on the skin’s surface. Hence, the second principal component was interpreted as a comprehensive indicator of the location where pruritus originates. Categorizing the site of onset into mucous membranes and skin surfaces was deemed to have clinical significance. This classification holds importance in the realm of drug-induced rashes, where the involvement of mucous membranes at the onset site has been noted as a metric for clinicians to gauge the severity of the condition [39]. Since rashes are often accompanied by intense pruritus, instances where pruritus involves mucous membranes might warrant particular attention.

As for the third principal component, the vector representing adverse effects was likewise segregated into those with positive and negative correlations. Adverse reactions that exhibited strong negative correlations encompassed sensations such as itching at application sites, injection sites, and infusion sites. These side effects appeared to be more intrusive, primarily due to the use of needles in the administration process. However, preparations that induce pruritus at the application site do not typically involve the use of injection needles. Conversely, the side effects exhibiting a robust positive correlation included general itching, eye itching, and erythematous itching. Scratchiness is an encompassing side effect that incorporates the other Preferred Term (PT) denoted as pruritus within this study. Therefore, it was presumed that this side effect might cover cases that could be categorized under different PT terms. Notably, these side effects appeared to be less invasive, given that the administration method did not entail the utilization of needles. Consequently, the third principal component could be construed as an indicator of invasiveness in the method of administration, manifested as a positive or negative direction. While there could be clinical significance investigating the connection between drug-induced pruritus and the method of administration, a limited literature on this matter exists. Presently, the method of administration is reported as one of the risk factors associated with the development of disease in the context of a penicillin allergy. From an epidemiological standpoint, it is suggested that a parenteral administration is more likely to cause allergies compared to oral administrations. In this study, a penicillin allergy includes IgE-mediated reactions, pruritus, and/or nonurticarial skin erupions. However, it remains plausible that this observation stems from an oversight in accounting for the influence of dosage adjustments [40].

Drawing from these outcomes, a hierarchical cluster analysis was conducted to further elucidate the relationship between each primary ingredient and the respective drug.

### 3.5. Hierarchical Cluster Analysis

Utilizing a hierarchical cluster analysis, the dataset was partitioned into distinct groups sharing similarities. In this investigation, a total of 200 drugs underwent a division based on their correlation with the three principal components, followed by an assessment of the traits exhibited by these clusters.

An a priori principal component analysis reduces the dimensionality of the data and improves their explanatory power. Clusters with positive correlations (in blue) with the first, second, and third principal components included systemic antibacterial (amoxicillin), obstructive airway disease (budesonide), antineoplastic (carboplatin), systemic antiviral (acyclovir), and immunosuppressive (cyclosporine) drugs. The data were also included in the list. The interpretation of the main component indices allowed us to interpret these drugs as having high rates of itch development, which tended to occur on mucous membranes, and a high likelihood of being administered in a less invasive manner.

Cluster 2 (in the orange box), which was positively correlated with the first principal component but negatively correlated with the second principal component, included systemic antibacterials (azithromycin), immunosuppressants (alemtuzumab), systemic antihistamines (cetirizine), antipruritics, anesthetics (diphenhydramine), and immune serum and immunoglobulin (human immunoglobulin). Based on the interpretation of the principal component indices, it was possible to interpret that these drugs carried a higher incidence of itching that tended to develop on the skin.

Cluster 3 (in green), which was positively correlated with the first and second principal components but negatively correlated with the third principal component, included other nerve agents (buprenorphine), analgesics (buprenorphine), anesthetics/antipruritic agents (lidocaine), and antihypertensives (ambrisentan). The data were also included in the list of drugs. The interpretation of the main component indices allowed us to conclude that these drugs have high rates of itch development, which tends to occur on mucous membranes, and a high likelihood of being administered in a highly invasive manner.

Cluster 4 (in the purple box), which was positively correlated with the first principal component but negatively correlated with the second principal component, included anti-anemia agents (iron), ophthalmic agents (ciprofloxacin), blood substitutes and perfusate (calcium chloride), antidiarrheals, intestinal anti-inflammatory agents and anti-infectives (ciprofloxacin), and systemic antibacterial agents (prednisolone). Based on the interpretation of the index of principal ingredients, these drugs could be considered to have higher rates of itch onset that were more likely to develop on the skin.

We examined the extent to which the drugs in these clusters were consistent with the interpretation of the principal components. However, to our knowledge, the relationship between the drugs that induce itching and the site of the onset of itching or the method of drug administration has not been fully elucidated. However, knowledge on the pathogenesis mechanism was accumulated.

The mechanism by which itching develops on mucous membranes was investigated. Itching appears in many forms of rhinitis [41]. This itch is transmitted by the ocular and maxillary nerves, which innervate the oral cavity, the lumen of the upper airway, and the conjunctiva of the eye, each of which transmits signals through epithelial cell junctions. These areas are innervated by TRP channels, which typically respond to a wide range of chemicals at concentrations approximately three orders of magnitude higher than most olfactory responses.

Based on the findings, we assumed that an itch tends to develop on mucous membranes when the drug acts on nerves innervating mucosal tissues. The drugs in Cluster 1 for obstructive airway disorders (budesonide) and in Cluster 3 for other nerve agents (buprenorphine), pharyngeal agents (buprenorphine), and ophthalmologic agents (lidocaine) tended to cause itching on mucous membranes because the drug application site was mucosal tissues or nerves.

The invasiveness of the administration method was evaluated according to the site of application of the itch-inducing drug. For drugs in Cluster 1, carboplatin was only administered intravenously, but the other drugs had routes of administration that did not involve the use of syringes; therefore, they tended to be less invasive. Conversely, for Cluster 3 drugs, buprenorphine and lidocaine were administered intravenously, whereas ambrisentan was administered orally; therefore, they tended to be more invasive.

### 3.6. Clinical Application

The findings derived from the adverse drug reaction spontaneous reporting database harbor numerous biases, as elucidated in the subsequent section on Limitations. Hence, prudent judgment is imperative in the clinical application of the insights garnered from this study. Proceeding from this premise, the ensuing recommendations are posited. Because the results of the volcano plot identified drugs that induce pruritus, it is desirable to avoid these drugs. Based on the results of principal component and hierarchical cluster analyses, we believe it will be possible to select drugs with low risks of pruritus in terms of both the site of onset and method of administration. The drugs in Cluster 3 should be avoided because they tend to cause pruritus on mucous membranes and their administration methods tend to be more invasive. However, these results and considerations were not consistent with previous study findings [41]. Prospective clinical trials are needed to utilize these findings. When substantiated, these findings will be useful for pharmacists to avoid itching when dispensing medications in clinical settings, including community practice, by collaborating with physicians and other healthcare practitioners.

### 3.7. Limitations

This study is subject to eight limitations arising from the database used.

Initially, the database comprises instances acknowledged as adverse events by the individuals submitting the reports. This is founded on the aggregation of spontaneous reports originating from healthcare professionals, pharmaceutical firms, and patients. Consequently, instances of repeated reports from numerous submitters are present within the database. Furthermore, despite the recommendation by FAERS for the reporting of all suspected adverse events, it is acknowledged that limitations exist due to potential challenges in diagnosing adverse events. Notably, approximately 66% of physicians who abstained from reporting indicated their non-reporting in instances where the causal connection between the adverse event and the suspected drug was ambiguous [42]. This recognition underscores that the under-reporting of adverse events stemming from such factors is a formidable challenge. Moreover, the reporting trends tend to vary over time. Emerging drugs may experience reduced reporting, while drugs garnering augmented attention in the context of adverse events may encounter elevated reporting rates. Furthermore, the number of reports and signals can be underestimated or overestimated because of multiple factors [40,43]. The “Weber effect” describes the phenomenon by which the number of adverse event reports increases during the first 2 years after a new drug is launched and then declines. Another is the “notoriety effect,” in which reporting increases after a drug-related adverse event is highlighted. In addition, a “spillover effect” can occur, whereby the same class of drugs might also be reported more frequently because of the notoriety effect. In addition, it has been reported that the association of certain adverse events with other drugs can weaken the signal detected imbalance analysis. This is categorized as a “masking effect” or “cloaking effect.” The presence of these biases poses challenges in accurately determining the denominator information for drug-induced pruritus, i.e., the total number of individuals using the drug [44,45]. In our endeavor to appropriately evaluate the provocation of adverse events for each drug, we employed an imbalance analysis strategy. This approach detects a signal, represented by the ROR, which capitalizes on the imbalanced risk of adverse events for individual drugs, enabling a relative assessment [46]. Additionally, to ensure the signal’s reliability, we ensured that each case exhibited a significant *p*-value and featured a minimum threshold of reported instances.

Secondly, in certain instances, cases of pruritus induced by drugs are misdiagnosed as non-drug-related pruritus, leading to incorrect diagnoses of new diseases. In such cases, H1 antihistamines, the first-line drugs for pruritus, were commonly prescribed, although the causative drug needed to be discontinued. H1 antihistamines were not effective, and patients who did not respond to these drugs were switched to other classes of antipruritic drugs. Thus, when the cause of pruritus was not ascertained to be a drug, a new drug was prescribed. This medical phenomenon is referred to as a “prescribing cascade,” in which the side effects of one drug were erroneously diagnosed as a new disease, resulting in the prescription of yet another new drug [47]. When occurring repeatedly, this process resulted in patients taking numerous unnecessary medications, which could lead to additional health problems. Because pruritus is a complex condition with many unexplored aspects, the risk of exacerbation attributable to the prescription cascade awaits further collection and an analysis of more detailed medical information.

Third, this study did not control for confounding factors by multivariate or multiple logistic analysis. This is because these analyses are not common in studies using adverse drug reaction databases. However, we believe that considering confounding factors is an issue for future studies.

Fourth, we did not perform an analysis controlling for drug interactions against the adverse drug reaction database. Drug interactions could increase drug plasma concentrations and lead to adverse events. Additionally, drug duplication and medication errors could have led to the occurrence of adverse events.

Fifth, the FAERS database used in this study did not register pruritus severity or complications. We recognize that this is one of the limitations of adverse drug reaction databases. Therefore, it is expected that future studies segregate cases by severity or complications.

Sixth, we did not limit the number of patients analyzed by attribute. This was done to ensure that a large number of patients were included in the analysis and to stabilize the ROR results using the adverse drug reaction database. Thus, it is a common practice to include all patients. In contrast, some studies used methods to classify and stratify the patient population to limit the number of subjects or collect more detailed information. In this study, stratification was not performed because the objective was to comprehensively analyze the entire population in terms of their propensity to itch. We believe that this is an issue for future research.

Seventh, the feasibility of the analysis varies depending on the specific context. Cases involving multiple drugs make it challenging to pinpoint the exact drug responsible for the adverse event [48]. While fatal adverse events undergo verification by the FDA, other adverse events are entered based on the reporter’s discretion, potentially leading to suspected adverse events being false positives.

Eighth, while this analysis constitutes a risk assessment for patients globally reporting adverse reactions, an inherent bias exists in the reporting frequency across different countries [49]. Hence, future endeavors should undertake comprehensive studies encompassing diverse or restricted populations, considering variations in patient demographics like ethnicity and medical history.

## 4. Materials and Methods

### 4.1. Data Source

In this investigation, the FAERS database was employed, which constitutes the US FDA repository of reports concerning adverse events arising from globally used medications, encompassing Japan as well. Notably, this database boasts a higher accumulation of aggregated cases in comparison to other repositories of adverse event reports [45]. The dataset comprises inputs from healthcare professionals and spontaneous reports furnished by patients. Sakaeda, in 2013, demonstrated the utility of data mining algorithms in detecting signals within the FAERS dataset, with the ROR emerging as the most prominent indicator [45]. To commence, the FAERS database was procured at no cost from the FDA’s official website [50]. Subsequently, data cleansing procedures were executed, yielding a refined database tailored for analytical purposes. The study extracted a substantial count of 11,810,863 adverse drug events recorded from the first quarter of 2004 up to the initial quarter of 2020. Because this study used anonymized data from an open-access database, the requirements for ethical approval and informed consent were waived by the Ethics Committee of the Meiji Pharmaceutical University.

### 4.2. Data Cleaning

In this study, duplicate cases were eliminated. Within each data table, instances with duplicate combinations of values in columns necessary for the analysis were filtered out, retaining only cases with distinct value combinations in the requisite columns. In simpler terms, solely cases featuring distinct value combinations in columns essential for analysis were extracted. The indispensable columns for analysis encompassed the patient ID and adverse event date in the Demographic table; the patient ID, drug name, and drug-specific number of the case in the Drug table; the patient ID, primary disease, and drug-specific number of the case in the Indication table; the patient ID and primary disease in the Reaction Patient ID and the name of the adverse event in the Reaction table; the patient ID and unique drug number of the case in the Therapy table; and the initiation date of the medication and the date of the onset of the adverse event in the Therapy table.

### 4.3. Definitions of Pruritus-Related Adverse Events

The adverse event descriptors found within the FAERS database originated from the System Organ Class (SOC) delineated in the Medical Dictionary for Regulatory Activities (MedDRA), an international compendium of pharmaceutical terminologies. These SOCs serve as an integral component of the MedDRA hierarchy and constitute the uppermost tier of concepts that indicate the organs wherein the fundamental PT expressions are categorized. Every PT is associated with one or more corresponding SOC [51]. In the present investigation, PTs associated with pruritus were exhaustively extracted from MedDRA version 23.0. Specifically, terms containing “prurtus”, “pruritic”, “itch”, or “itching” were considered. Among these, PTs encompassing 37 terminologies implying itching were classified as “pruritus”, which constitutes the term under scrutiny within this study (Table 4).

### 4.4. Preparation of Data Table for Analysis

FAERS comprises seven distinct data tables: (1) Demographic table, (2) Drug table, (3) Indication table, (4) Outcome table, (5) Reaction table, (6) Report sources table, and (7) Therapy table [52]. Figure 1 shows the items included in each table and the number of reports from Q1 2004 to Q1 2020.

In this study, the analytical data tables were constructed following the methodology outlined by Kurosaki et al. [53].

After the removal of incomplete data entries from each respective data table, the unique identification numbers of primary reports were cross-referenced against a demographic table, featuring 11,810,863 reports, a Drug table, encompassing 75,403,849 reports, a Therapy table, housing 40,164,871 reports, and an Indication table, accounting for 25,929,031 reports. These tables were amalgamated into a unified data table while eliminating instances of duplicated reports [54]. The exclusion of these cases reduced biases. Prior studies reported that de-duplication improves data reliability.

Subsequently, for the purpose of a chronological arrangement, we gathered reports where the onset of adverse events aligned within the timeframe of medication usage. These reports were then consolidated into a Reaction table, resulting in a total of 35,393,413 entries. However, considering that drugs listed in the Drug table encompassed designations as suspect drugs, concomitant drugs, or interactions based on their involvement in adverse drug reactions, all of these categories were regarded as prospective suspect drugs. Throughout the time series, we successfully extracted 8,184,203 reports illustrating a discernible connection between drug utilization and adverse drug reactions. It is worth noting that instances where drug indications were also reported as adverse events, typically due to insufficient drug efficacy, could potentially introduce spurious signals. To counter this, we meticulously identified reports where pruritus-related PTs were cited as indications (underlying diseases). Subsequently, 18,242 reports involving patients in this context were systematically removed based on case IDs. Although the potential for the exclusion of non-spurious signals could not be completely ruled out, its influence on the computation of the ROR employed for analysis was minimal. The resulting dataset, comprising 8,165,961 reports post-elimination, constituted the foundation for the analysis.

### 4.5. Patient Information on Drug-Induced Pruritus

A bivariate analysis was executed employing patient particulars, such as gender, age, and weight, as explanatory factors and itchiness as the dependent variable. Fisher’s exact test was employed for gender, a categorical variable, while Wilcoxon’s rank sum test was applied for age and weight, being continuous variables, with corresponding *p*-values calculated. Additionally, the dataset was stratified based on gender, and for each group, the count of cases/patients, mean age accompanied by standard deviation, and mean weight along with standard deviation were computed, contingent upon the presence or absence of pruritus. However, prior to conducting these analyses, any missing values or outliers were effectively removed. In terms of age, values ranging from 0 to 130 years were retained, while all other values were identified as outliers. Similarly, for weight, values between 0 kg and 600 kg were retained, whereas all other values were categorized as outliers.

### 4.6. Extraction of Suspect Drugs for Pruritus Using Data Mining Methods

Signal detection within the context of a spontaneous adverse event report database constitutes a data mining approach that facilitates the inference of connections between pharmaceutical products and adverse events. This is achieved even in cases where the precise number of patients utilizing a specific medication (population) remains unknown through the exploitation of disproportionate and relative occurrences of each adverse event [45]. This methodology enables the calculation of signal indicators. For this study, the ROR was employed, which is recognized as a more sensitive and less biased signal measure compared to the proportional reporting ratio.

**Figure 6:**
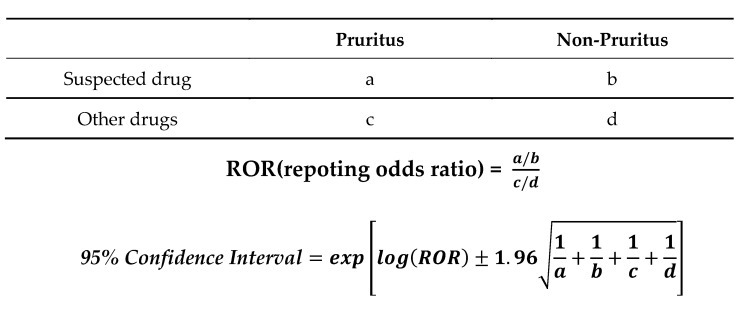
Cross-tabulation table and formula for the reporting odds ratios (RORs) reported for adverse events.

The analysis encompassed all drugs categorized by their generic names within the FAERS database. The analysis dataset was organized based on the presence or absence of pruritus for each drug, subsequently forming a cross-tabulation table (Figure 6). Given that a 2 × 2 contingency table cannot be accurately calculated with cells containing zero values, and small cell frequencies introduce instability in estimation, a correction was applied by adding 0.5 to all cells (Haldane Anscombe semi-correction) [55]. Subsequently, the ROR was computed (Figure 6). This ratio represents the probability of a specific drug triggering a particular adverse event in relation to the probability of another drug causing a distinct adverse event (Figure 2) [45]. Signal presence was defined based on the lower limit of the 95% confidence interval of the ROR surpassing 1, determined through *P* values from Fisher’s exact test. Ultimately, a combination of the ROR and *p*-value statistics facilitated the assessment of the proportion and significance of pruritus for each drug, visualized through a volcano plot. This method is widely recognized in the realm of bioinformatics and genomics for visualizing trends in gene expression [56].

For this study, drugs exhibiting a strong correlation with drug-induced pruritus were designated as those possessing a ROR of ≥1, a Fisher’s exact probability test *p*-value < 0.05, and a minimum of 50 reports. These drugs were identified as demonstrating a signal associated with occurrences of induced pruritus.

The cells within the cross-tabulation encompass four distinct groups: the cohort using the suspected drug, the cohort utilizing other medications, the group experiencing pruritus, and the group not encountering pruritus (where “a” through “d” denote the respective report counts). Utilizing the mentioned formulas, the RORs alongside their corresponding 95% confidence intervals were derived.

### 4.7. Analysis of Drugs Associated with Pruritus Using Principal Component Analysis and Hierarchical Cluster Analysis

To examine the similarity in the relationship between each drug and pruritus, this study employed a principal component analysis and a hierarchical cluster analysis. A hierarchical cluster analysis of the principal components was performed by applying a previously described method [57,58,59]. Adverse event descriptions from FAERS were organized into groups according to disease state and domain using a standardized MedDRA query (SMQ). Initially, 29 terms were extracted from the PT category of the MedDRA query designed for pruritus, with a minimum reported patient count (N) of 50 (Table 4). Subsequently, a 2 × 2 contingency table was generated for each of these 29 adverse event terms, contrasting the presence or absence of all medications reported in FAERS. Based on these tables, the ROR was computed. The calculated RORs were then transformed into natural logarithms and consolidated into a single dataset, with each adverse event term along the *X*-axis and each medicinal product along the *Y*-axis. This dataset was constructed specifically for the 200 drugs with over 10,000 adverse event reports.

A principal component analysis was conducted on this dataset using a covariance matrix. This analytical method allows for the examination of quantitative data described by multiple variables by reducing correlations between variables and condensing them into a smaller number of composite variables, while minimizing information loss [60]. The contribution rates and factor loadings for each variable were computed for each principal component. The first, second, and third principal components were used to interpret the characteristics and side effects of the medications.

Additionally, a hierarchical cluster analysis was applied to visually group the adverse event terms. A hierarchical cluster analysis is an analytical technique where clusters are sequentially formed from highly similar data, visualized in a tree diagram. In this study, the Ward method, utilizing Euclidean distance with loadings derived from the principal components, was employed. The hierarchical cluster analysis resulted in the creation of three distinct clusters. Clusters were created where the slope of the distance graph increased rapidly (Figure 5). This hierarchical cluster analysis established six clusters.

### 4.8. Statistical Analysis

All statistical analyses were carried out using JMP Pro 16.2.0 (SAS Institute Inc., Cary, NC, USA), and statistical significance was determined based on a *p*-value threshold of less than 0.05.

## 5. Conclusions

This study aimed to analyze drug-induced pruritus by utilizing the FAERS database, which contains adverse event reports from various regions worldwide. Notably, this study stands as the first comprehensive effort to identify causative drugs and assess their relative risk. However, the findings in this study were obtained for drugs with high rates of use by analyzing drugs with >50 reported cases. Because of the reduction in bias, new findings on the characteristics and trends of causative drugs from this study are likely to be applicable to many other drugs. These findings highlight the drugs associated with the induction of pruritus, which will allow us to avoid pruritus as a side effect in advance when selecting a drug. In addition, because we developed a new classification method for classifying suspect drugs for pruritus according to the site of pruritus onset and the method of administration, we believe that these findings will be useful in clarifying the mechanism of drug-induced pruritus onset. Understanding the pathogenesis of pruritus could lead to the development of targeted prevention and treatment strategies, ultimately enhancing the quality of life for affected patients.

## Figures and Tables

**Table 1:** Characteristics of the patients in the data tables.

		Pruritus		Non-Pruritus		Sum	*p*-Value ^a^
		Average ± SD	Number of Cases	Average ± SD	Number of Cases	Number of Cases	
All data table	Both gender	-	86,989	-	7,309,045	7,396,343	
	Age	50.30 ± 20.24	81,230	53.17 ± 21.09	6,760,759	6,841,989	<0.01
	Weight	88.26 ± 44.93	39,102	83.44 ± 44.55	3,821,673	3,860,775	<0.01
Male data table	Age	52.13 ± 20.65	26,081	55.57 ± 21.11	2,726,069	2,752,150	<0.01
	Weight	91.27 ± 44.77	13,804	86.22 ± 45.34	1,566,742	1,580,546	<0.01
Female data table	Age	49.44 ± 19.99	55,149	55.15 ± 20.92	4,034,690	4,089,839	<0.01
	Weight	86.63 ± 44.94	25,298	81.87 ± 43.90	2,254,931	2,280,229	<0.01

Data are presented as the mean ± standard deviation in each category. Prior to conducting these analyses, any missing values or outliers were effectively removed. ^a^ Wilcoxon’s rank sum test.

**Table 2:** Drugs with signals associated with induced cases of pruritus.

Therapeutic Correspondence	Drug
Ophthalmologicals	Acetylcysteine, azithromycin, besifloxacin, bimatoprost, boric acid, brimonidine, brinzolamide, bromfenac, carmellose, cefuroxime, chlorhexidine, clonidine, desonide, diclofenac, dorzolamide, erythromycin, fluocinolone acetonide, fluorescein, fluorometholone, glycerol, hypromellose, ketotifen, latanoprost, lifitegrast, liquid paraffin, loteprednol, macrogol 400, moxifloxacin, naphazoline, neomycin, netarsudil, olopatadine, paraffin, polymyxin b, polysorbate 80, povidone–iodine, propylene glycol, sulfacetamide, tetracaine, tetracycline, tetryzoline, timolol, tobramycin, travoprost, triamcinolone, and vancomycin
Antibacterials for systemic use	Amoxicillin, azithromycin, bacitracin, cefaclor, cefadroxil, cefalexin, cefazolin, cefdinir, cefixime, cefoxitin, cefprozil, cefradine, ceftaroline fosamil, ceftazidime, ceftibuten, ceftizoxime, ceftriaxone, cefuroxime, clarithromycin, clindamycin, cloxacillin, dalbavancin, dicloxacillin, doxycycline, erythromycin, and fosfomycin, gemifloxacin, metronidazole, moxifloxacin, nafcillin, neomycin, oritavancin, phenoxymethylpenicillin, piperacillin, polymyxin b, pristinamycin, sulfamethoxazole, tazobactam, tetracycline, ticarcillin, tobramycin, trimethoprim, and vancomycin
Contrast media	Amidotrizoic acid, barium, gadobenic acid, gadobutrol, gadodiamide, gadofosveset, gadopentetic acid, gadoteric acid, gadoteridol, gadoxetic acid, iodixanol, iohexol, iomeprol, iopamidol, ioversol, ioxaglic acid, ioxilan, and perflutren
Antifungals for dermatological use	Butenafine, ciclopirox, clotrimazole, econazole, efinaconazole, fluconazole, ketoconazole, miconazole, nystatin, naftifine, selenium sulfide, tioconazole, terbinafine, and tavaborole
Other dermatological preparations	Brimonidine, diclofenac, dupilumab, eflornithine, hydroquinone, ivermectin, minoxidil, sulfur, pimecrolimus, and povidone–iodine
Corticosteroids, dermatological preparations	Beclometasone, clobetasol, desoximetasone, desonide, fluorometholone, fluocinonide, fluocinolone acetonide, methylprednisolone, triamcinolone, and ulobetasol
Gynecological anti-infectives and antiseptics	Ciclopirox, clindamycin, clotrimazole, econazole, ketoconazole, metronidazole, miconazole, nystatin, povidone–iodine, and tioconazole
Antineoplastic agents	Aminolevulinic acid, asparaginase, carboplatin, celecoxib, cetuximab, chlormethine, oxaliplatin, pegaspargase, rituximab, and tretinoin
Immunosuppressants	Anakinra, belimumab, dalimumab, etanercept, fumaric acid, ixekizumab, ocrelizumab, sarilumab, and secukinumab
Vaccines	HPV vaccine, pneumococcal vaccine, and varicella zoster vaccine

**Table 3:** Drugs positively correlated with the first principal component obtained by a principal component analysis and their drug classification.

Therapeutic Correspondence	Drug
Antibacterials for systemic use	Amoxicillin, ceftriaxone, ciprofloxacin, clarithromycin, levofloxacin, linezolid, metronidazole, moxifloxacin, piperacillin, sulfamethoxazole, tazobactam, trimethoprim, and vancomycin
Antineoplastic agents	Carboplatin, celecoxib, gemcitabine, imatinib, nilotinib, nivolumab, oxaliplatin, pazopanib, regorafenib, and rituximab
Ophthalmologicals	Aciclovir, azithromycin, ciclosporin, ciprofloxacin, diclofenac, levofloxacin, lidocaine, moxifloxacin, and vancomycin
Immunosuppressants	Alemtuzumab, certolizumab pegol, ciclosporin, fumaric acid, infliximab, natalizumab, teriflunomide, tocilizumab, and tofacitinib
Antivirals for systemic use	Aciclovir, dasabuvir, emtricitabine, lamivudine, sofosbuvir, telaprevir, and valaciclovir

**Table 4:** Analysis of 37 PTs.

	Preferred Term	Number of Patients
1	Pruritus	40,618
2	Rash Pruritic	6287
3	Injection Site Pruritus	3034
4	Application Site Pruritus	2401
5	Eye Pruritus	2278
6	Vulvovaginal Pruritus	647
7	Oral Pruritus	400
8	Ear Pruritus	294
9	Eyelids Pruritus	263
10	Anal Pruritus	253
11	Pruritus Genital	195
12	Pruritus Allergic	127
13	Tongue Pruritus	124
14	Infusion Site Pruritus	122
15	Implant Site Pruritus	97
16	Nasal Pruritus	89
17	Lip Pruritus	80
18	Instillation Site Pruritus	46
19	Catheter Site Pruritus	23
20	Gingival Pruritus	14
21	Itching Scar	7
22	Administration Site Pruritus	6
23	Incision Site Pruritus	6
24	Vaccination Site Pruritus	6
25	Aquagenic Pruritus	5
26	Cholestatic Pruritus	4
27	Stoma Site Pruritus	2
28	Vessel Puncture Site Pruritus	2
29	Medical Device Site Pruritus	1
30	Popular Pruritic Eruption of HIV	0
31	Vascular Access Site Pruritus	0
32	Tumor Pruritus	0
33	Post-Procedural Pruritus	0
34	Puncture Site Pruritus	0
35	Uraemic Pruritus	0
36	Senile Pruritus	0
37	Brachioradial Pruritus	0

## Data Availability

Data is contained within the article.
